# Tracking capsule activation and crack healing in a microcapsule-based self-healing polymer

**DOI:** 10.1038/s41598-019-54242-7

**Published:** 2019-11-28

**Authors:** S. A. McDonald, S. B. Coban, N. R. Sottos, P. J. Withers

**Affiliations:** 1 Henry Royce Institute for Advanced Materials, Department of Materials, University of Manchester, Manchester, M13 9PL UK; 2 Centrum Wiskunde & Informatica, Computational Imaging Group, Science Park 123, 1098XG Amsterdam, The Netherlands; 3 Department of Materials Science and Engineering, University of Illinois at Urbana-Champaign, Urbana, IL 61801 USA

**Keywords:** Polymers, Composites, Characterization and analytical techniques, Imaging techniques, Phase-contrast microscopy

## Abstract

Structural polymeric materials incorporating a microencapsulated liquid healing agent demonstrate the ability to autonomously heal cracks. Understanding how an advancing crack interacts with the microcapsules is critical to optimizing performance through tailoring the size, distribution and density of these capsules. For the first time, time-lapse synchrotron X-ray phase contrast computed tomography (CT) has been used to observe in three-dimensions (3D) the dynamic process of crack growth, microcapsule rupture and progressive release of solvent into a crack as it propagates and widens, providing unique insights into the activation and repair process. In this epoxy self-healing material, 150 µm diameter microcapsules within 400 µm of the crack plane are found to rupture and contribute to the healing process, their discharge quantified as a function of crack propagation and distance from the crack plane. Significantly, continued release of solvent takes place to repair the crack as it grows and progressively widens.

## Introduction

Traditionally the development of engineering materials has been predicated on the design of new materials with greater damage tolerance, alongside the development of non-destructive evaluation methods for their inspection such that the part can be withdrawn from service prior to failure. By contrast, inspired by nature, self-healing materials are a relatively new and underexplored class of materials that exhibit the ability to repair themselves and to recover their functionality^1^. Certain self-healing materials have the ability to partially, or completely, repair cracks that may arise in service, such that the original properties are fully or partially recovered. Self-healing of damage can therefore extend the lifetime and reliability of the material/component. This can be particularly useful in cases where repair or replacement might be difficult or hazardous, or where frequent inspections are difficult. In this respect applications range from oil and gas structures, to aerospace polymer composites, through space applications, to corrosion resistant coatings and even to medical implants.

**Figure 1:**
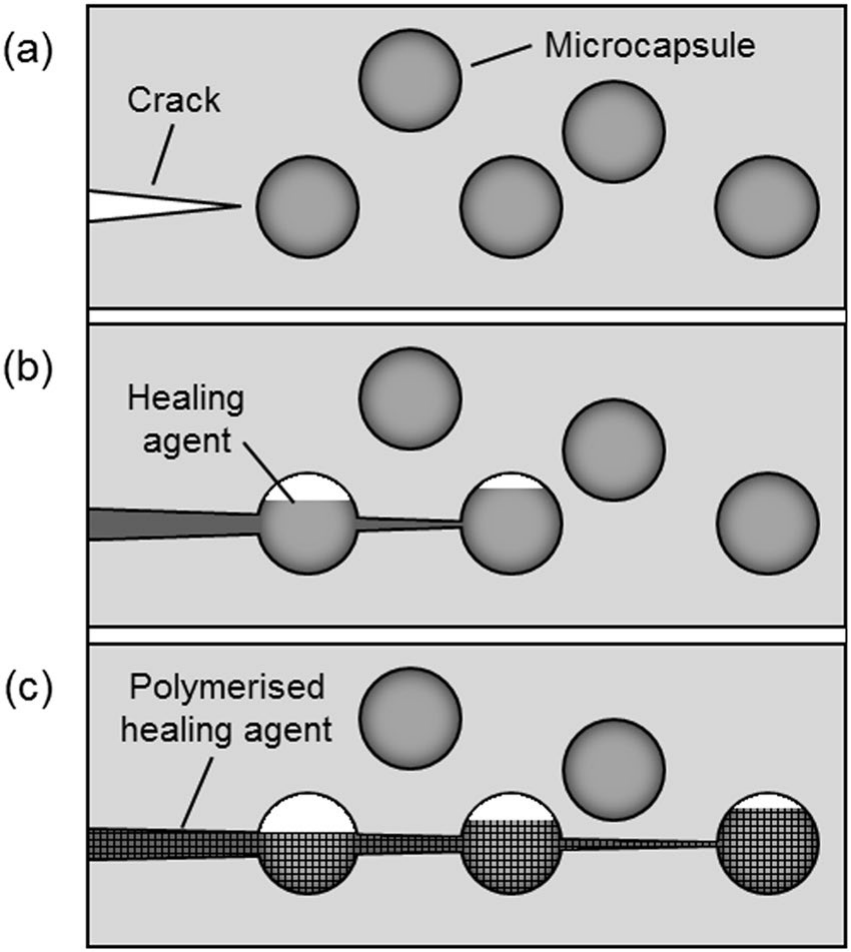
Schematic of the self-healing process using embedded microcapsules. (**a**) A crack forms in the matrix due to damage; (**b**) the growing crack ruptures microcapsules in its path, thereby releasing the healing agent into the crack plane; (**c**) polymerization occurs and the crack faces are bonded closed^3^.

While various strategies (capsules, vascular and intrinsic healing) have been developed to deliver self-healing for different material classes (metals, ceramics, polymers), all rely on a mobile phase or reactive healing agent which can repair the crack. This is triggered either by the occurrence of damage itself (the ideal case, defined as autonomic self-healing), or by external stimuli (non-autonomic self-healing) such as heat or light or targeted triggers such as a laser beam, or inductive or resistive heating^1,2^. After healing, the repair should have properties approaching that of the undamaged material, retaining structural integrity and extending the lifetime of the material. For structural polymeric materials one of the most successful approaches has been to incorporate microcapsules that are filled with a liquid healing agent (see Fig. 1)^3^. When a crack propagates through the material the healing agent is released and becomes the mobile phase. Polymerization of the healing agent is triggered by contact with an embedded catalyst, resulting in a bonding of the crack faces.

The occurrence of self-healing in microcapsule based polymer composite systems has been demonstrated using fracture experiments^3–6^, showing as much as 75% recovery of the original fracture toughness and a high healing efficiency reaching values of up to 90%. The average critical fracture load required to propagate a crack in an undamaged self-healing polymer was found to be 20% higher than the average value for neat epoxy samples containing no microcapsules or catalyst^3^. The inherent toughness of the epoxy is therefore shown to increase with the addition of microcapsules/catalyst. This microcapsule induced toughening has been observed to have a strong dependence on the size and density of the embedded microcapsules^4^. Furthermore, the presence of the microcapsules has been shown to significantly decrease the fatigue crack growth rate and increase the fatigue lifetime (more than 100 times increase at low *K*/*K*_1c_ ratios)^5^. Given that the microcapsules impact on the stiffness and strength of the polymer it is essential to optimize their size, spacing and activation to effect healing at microcapsule loadings that are as low as possible.

Establishing a 3D picture of how a crack interacts with the microcapsules in its path as it grows, in terms of how dense and how near the capsules must be to the crack in order to be activated and how effectively the capsules release the solvent, is therefore important for understanding and the subsequent optimization of this new class of materials in terms of activation of the healing process. This is especially important because, as stated in the introduction, many of the most potent applications are cases where this self-healing capacity is advantageous because inspection, repair or replacement are difficult. In this regard, X-ray computed tomography (CT) has become a powerful tool for imaging the spatial and temporal evolution of microstructures in three-dimensions (3D) across a wide range of scales and within a variety of engineering materials^7^ including during failure processes^8^. With regard to self-healing materials, X-ray CT has enabled the study of crack healing in MAX-phase ceramics^9,10^. Using the high flux of synchrotron X-rays the temporal evolution of local crack opening and repeated healing during multiple cracking and autonomous repair cycles was captured at a temperature of 1500 K^9^. In the context of capsule based self-healing, X-ray CT has previously been used post mortem to examine the extent of microcapsule activation^11^. Quantitative information was collected on the distribution of full and empty 60 µm capsules in the vicinity of the crack. The zone of ruptured microcapsules extended to ~75 µm each side of the crack surfaces. While post mortem studies are insightful they provide little information regarding the sequence of fracture events or the efficacy of the fluid in filling the crack as it progresses.

Here we use synchrotron X-ray micro-CT to follow *in situ* the growth of a crack in a self-healing material for the first time. The material comprises an epoxy matrix containing a microencapsulated reactive epoxy resin and solvent. Epoxies are versatile engineering polymers with excellent mechanical and chemical properties often used as matrices for polymer composites. They can undergo a low-temperature cross-linking reaction when discrete healing agents are introduced^2^. Upon rupture of the microcapsules and subsequent release of the solvent, the epoxy matrix swells and enables the resin to react with residual amines in the matrix.

A limitation of using conventional absorption contrast X-ray tomography is that the epoxy and the microcapsules have very similar X-ray absorption coefficients making the capsules difficult to differentiate from the matrix. Here we exploit phase contrast and an associated phase filter during reconstruction to significantly improve their detectability. The sample was mounted in a tension-compression mechanical testing rig which could be accommodated on the synchrotron X-ray beamline such that the filling of the crack could be monitored by X-ray CT during loading. This has allowed us to establish a quantitative picture as to the activation sequence over time; in particular how near the microcapsules must be to the crack, how they progressively release their contents, and the extent to which this fills and repairs the crack.

## Results and Discussion

**Figure 2:**
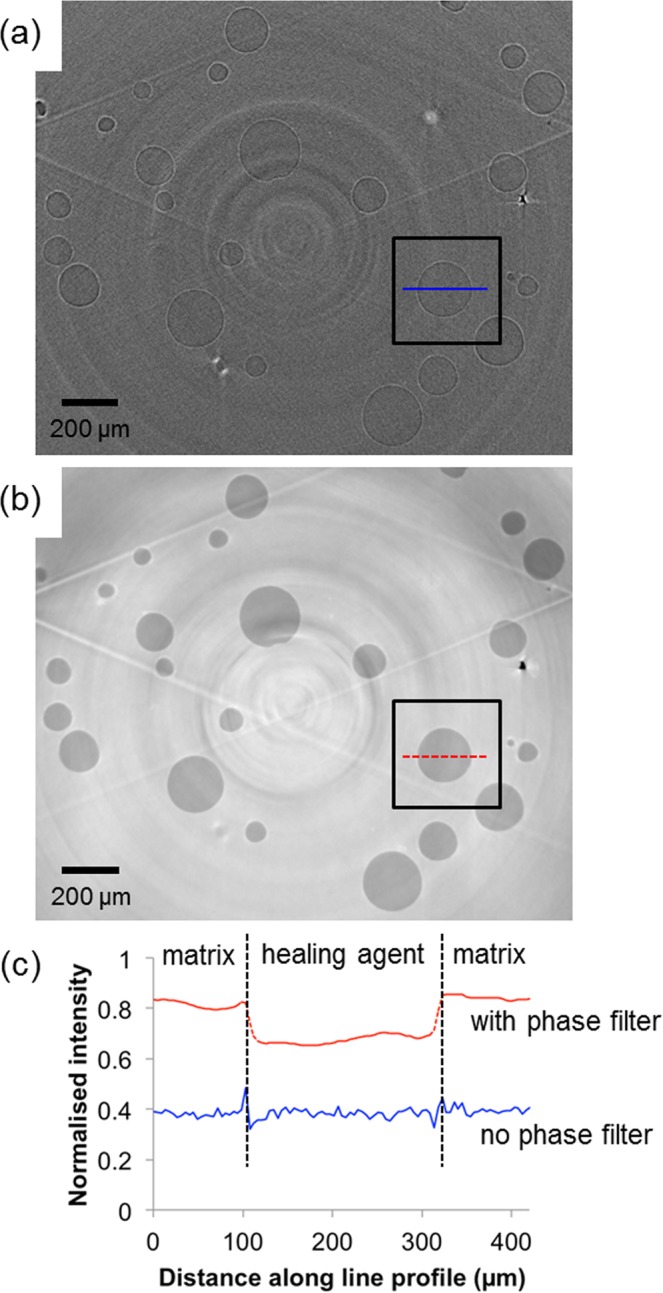
Demonstrating the application of phase contrast X-ray imaging. A virtual horizontal (*x-y*) slice through the reconstructed 3D volume of the microcapsule-based self-healing epoxy sample, shown (**a**) without phase backpropagation filtering and (**b**) with phase backpropagation filtering. (**c**) Line intensity profiles taken across the capsule indicated in (**a**,**b**), demonstrating the effect of the filter.

**Figure 3:**
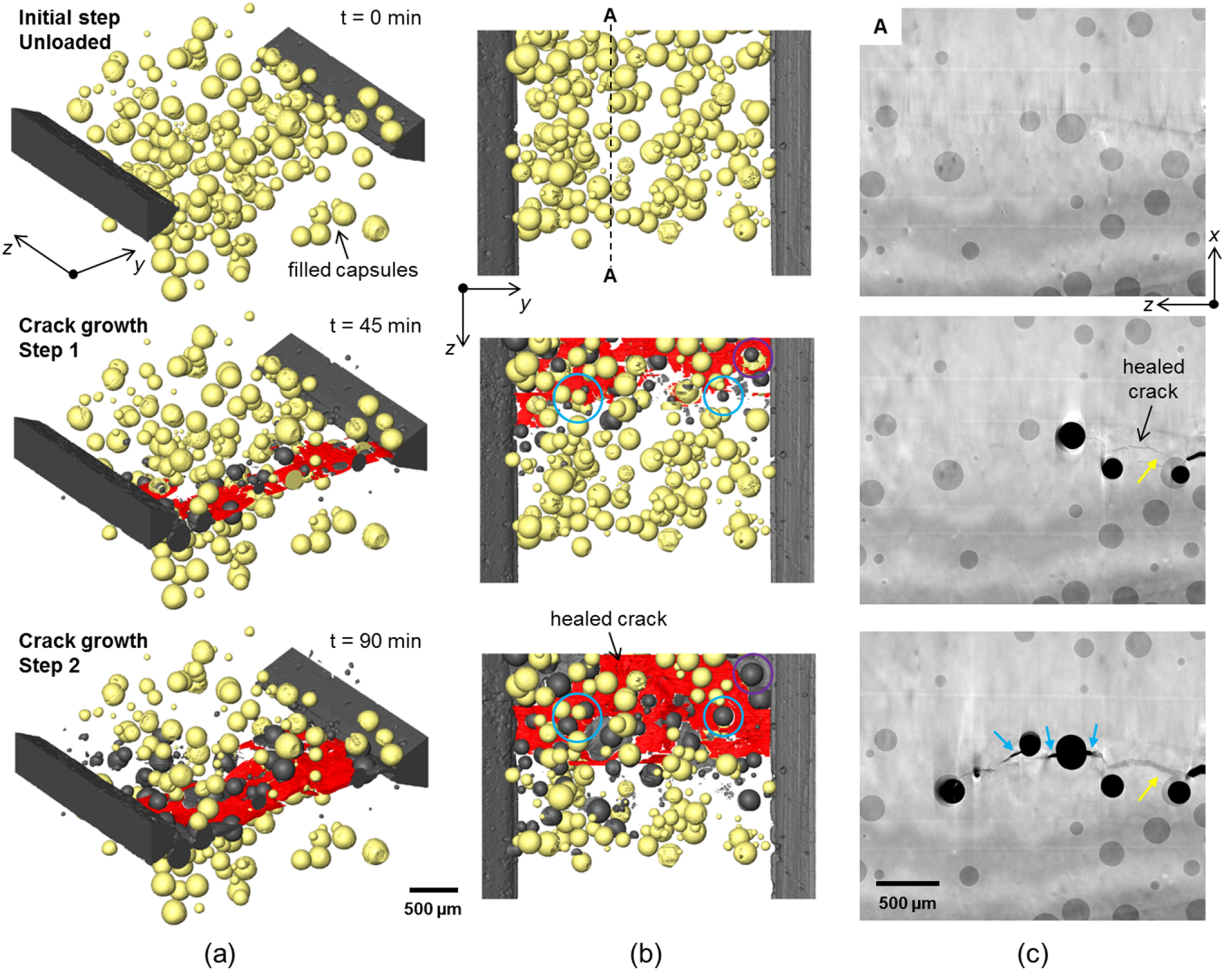
Tracking capsule activation, release of solvent and crack healing during propagation of the crack. (**a**) 3D perspective and (**b**) plan views of 3D surface renderings of the region of interest (2.2 × 3.4 × 2.7 mm^3^ (*x*, *y*, *z*)) showing the self-healing epoxy sample at several states during progressive loading to grow a crack. The matrix is rendered transparent, the filled capsules shown in yellow, empty capsules/crack in grey and the filled/healed crack in red. (**c**) 2D virtual greyscale slices intersecting the sample (and crack) at the location indicated ‘A’. The same equivalent slice is shown for each crack growth step. The black regions correspond to unfilled crack/capsules. The direction of crack growth is along ‘*z*’ (right to left). The time stated for each step represents the approximate time accumulated since the scan for the initial unloaded step was started.

As stated above, the epoxy matrix material and the healing agent contained within the microcapsules have very similar X-ray attenuation. An initial investigation was performed in order to determine, in terms of image contrast and signal-to-noise ratio, the level to which the capsule contents could be distinguished from the matrix. The improved differentiability between the matrix and healing agent constituents, through the application of a phase backpropagation filter (see Methods: X-ray microtomography), is demonstrated in Fig. 2. The 2D virtual sections in Fig. 2a,b illustrate the improvement in detectability achieved when a filter is applied during reconstruction. The bright and dark fringes at the capsule boundaries (see Fig. 2c) are minimized whilst enhancing the difference in greyscale intensity between the two materials and thereby facilitating their distinct segmentation. Ring artifacts and streak artifacts are evident in the reconstructed images in Fig. 2. The latter follow the edges of the sample along the triangular side grooves (outside of the region-of-interest of the images in Fig. 2, but shown in grey in the 3D surface renderings in Fig. 3). Their origin relates in part to the transition between sample and air where refraction (and thus phase shift) is especially high.

The rupture of the microcapsules and the release of the self-healing solvent are clearly shown in Fig. 3 as the crack propagates. The volume fraction of microcapsules in the region of interest is ~6%, while the average microcapsule diameter is ~150 µm (standard deviation: 61 µm). These values were determined from the segmented CT volume prior to cracking. The transition of capsules from filled (yellow) to empty (grey) as the crack propagates is evident. As the crack length increases between the two steps the extent of ruptured capsules along the crack growth direction (*z*) increases accordingly. It is important to note that for both crack growth steps capsules are observed to have ruptured ahead of the apparent filled (repaired) crack tip region (shown in red). It is believed this is because the spatial resolution of the technique means that it is difficult to detect and segment the crack very near its tip (see Fig. 3c). The microcapsules are observed to release their contents into the crack path. Many of the capsules fractured by the crack after the first step do not fully empty immediately, as indicated by the fact that some empty (broken) capsules increase in size from the first step to the second (see the examples circled in blue in Fig. 3b and the yellow arrow in Fig. 3c). It is also evident that some regions of the crack are not repaired (see for example the region of the crack neighbouring the two empty capsules in Fig. 3c indicated with blue arrows). Other regions have opened further but have been repaired by further discharge from the ruptured capsules (see the crack near the yellow arrow in Fig. 3c). It is noted also that there is a region near the mouth of the crack that was initially filled with solvent after the first crack growth step but upon further crack growth becomes unfilled (see the region circled in purple in the upper right corner in Fig. 3b). The microcapsule local to this region has emptied from the first step to the second. A possible explanation for this is that the crack gap here is too wide and there isn’t enough solvent available to fill and bond the crack faces.

**Figure 4:**
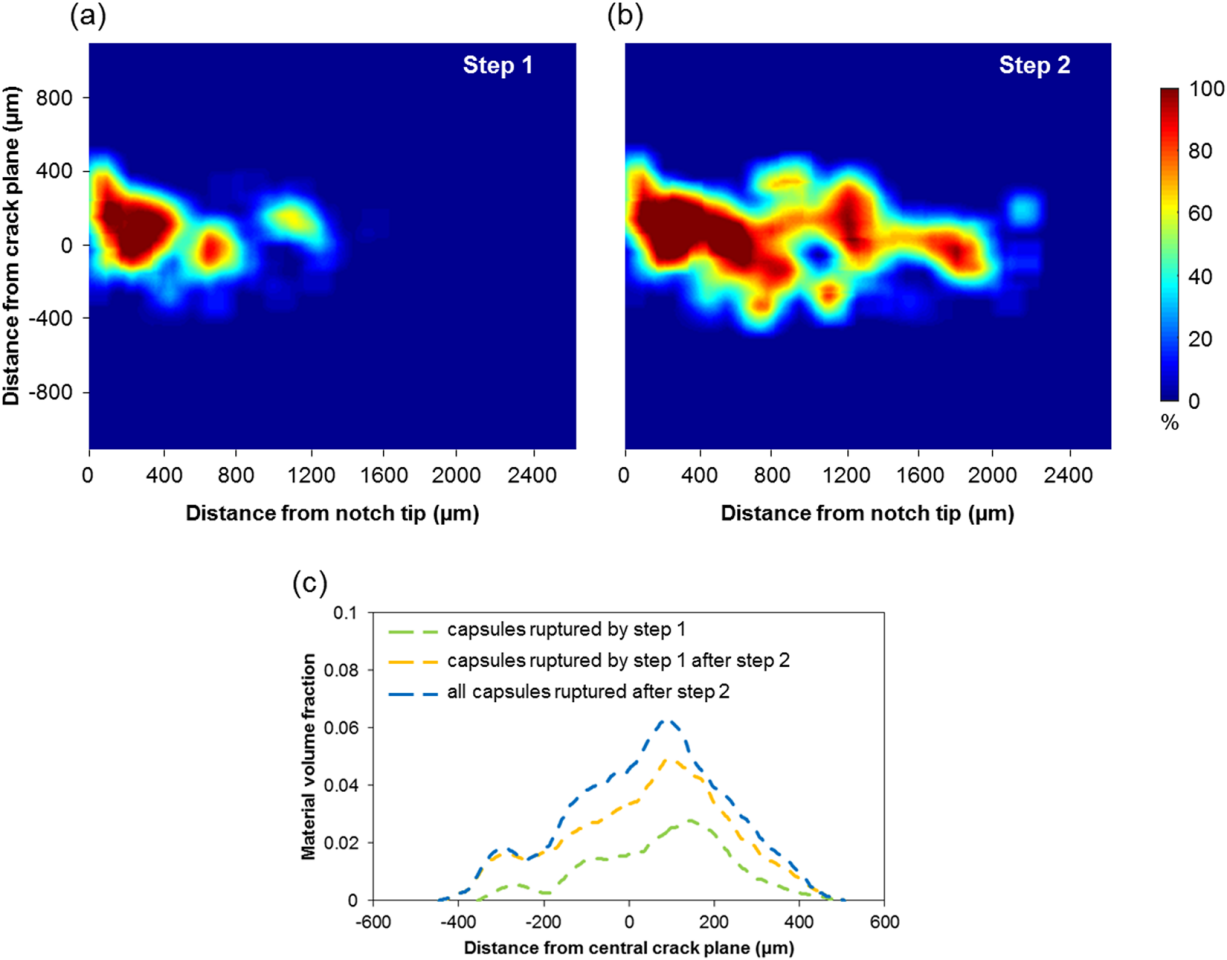
Quantifying the release of solvent into the crack. Percentage solvent released from capsules as a function of position after (**a**) step 1 and (**b**) step 2, integrated through thickness (*y* direction), and (**c**) the amount of solvent released as a function of distance from the crack plane by the capsules ruptured by step 1 and step 2.

In segmenting the 3D tomography reconstructions, quantitative analysis related to the release of solvent can be performed, shown in Fig. 4. The percentage of solvent released as a function of *x-z* position, from all microcapsules present through the *y*-direction, is shown in Fig. 4a,b. After the first crack growth step, near and either side of the crack plane, a region develops comprising ruptured capsules (see Fig. 4a). This zone of ruptured capsules extends over a distance across the crack plane of ~840 µm (~5 × the mean capsule diameter). Upon further crack growth (step 2) the number of filled capsules decreases further with the zone of ruptured capsules increasing slightly to a distance of ~950 µm across the crack plane (see Fig. 4b). It is also evident from Fig. 4c that some of the capsules ruptured by step 1 have released further solvent into the crack during step 2. This observation is important because it reveals that as the crack is further opened, there is still sufficient solvent to repair the growing crack. This result also confirms the earlier observation in Fig. 3 of the gradual emptying of individual capsules.

By quantifying the volume of empty capsules for each crack growth step a measure of the amount of fluid released into the crack can be approximated. This gives the total volumes of solvent released as 0.86 × 10^8^ µm^3^ (0.09 µl) and 2.32 × 10^8^ µm^3^ (0.23 µl) after steps 1 and step 2 respectively. The total volume released by the initially ruptured capsules after the second step is 1.82 × 10^8^ µm^3^ (0.18 µl), i.e. double the amount discharged by the first step. The capsules newly ruptured after the second step are distributed primarily in a region ± 200 µm from the central crack plane (i.e. comparing the yellow and blue curves in Fig. 4c). Naturally, the increase in the number of ruptured capsules, and thus fluid released, is related to the increase in crack length between the two steps which grows from ~1.4 mm to 2.3 mm as determined from Fig. 4a,b. One can predict the width of crack that can be repaired by the measured amount of solvent. For a triangular crack of 2.3 mm length and a crack mouth of 60 µm opening, both values being approximated from the length and opening of the crack in the current study after the second growth step, 0.23 µl of released solvent can repair a crack ~3.4 mm in width. Were the crack to grow longer or the mouth to open wider then the self-healing capability of the material would be exhausted.

The growth of a crack and its subsequent repair brought about by the release of solvent from ruptured microcapsules into the crack plane has been observed *in situ* in 3D using time-lapse X-ray tomographic imaging. Depletion of the microcapsules has been quantified for two crack growth steps, revealing the gradual release of liquid into the crack. While a comparison to control material (i.e. neat epoxy matrix containing no microcapsules, epoxy matrix containing empty microcapsules) is outside the scope of this study, Brown *et al*. performed fracture tests comparing self-healing and control samples^4^. Brittle fracture of the neat epoxy was observed and exhibited a mirror fracture surface, while the addition of microcapsules produced a transition of the fracture plane morphology to hackle markings. Increased hackle marking and subsurface microcracking were the operative mechanisms proposed giving rise to increased toughening associated with fluid-filled microcapsules. In control samples – neat epoxy and epoxy with voids (i.e. empty microcapsules) – hackle markings were restricted to a small region near the crack tip. Work by Rule *et al*. has shown that the amount of healing agent delivered by microcapsules to a crack face scales linearly with microcapsule diameter for a given weight fraction of capsules^12^. Self-healing performance was found to reach a maximum level only when sufficient healing agent is delivered to entirely fill the crack volume, i.e. the required minimum size and weight fraction of microcapsules depends on the size of the crack that is being healed. In the current study microcapsule activation and healing has been demonstrated for a single sample in terms of the size and density of microcapsules. Rule *et al*. found that self-healing can be achieved with as little as 1.25 wt% microcapsules or with microcapsules that are smaller than 30 µm for small crack volumes (crack separations of only 3 µm)^12^. It is possible to design self-healing systems tailored to repair specific crack volumes through the relationship between microcapsule size and weight fraction. As outlined here, 3D characterization of the healing process can provide valuable information about crack volumes, amount of solvent released in relation to microcapsule size and fraction, etc., which can be fed directly into their design. It is clear, though, that once the microcapsules have been depleted then further local repair is not possible, i.e. local healing and regeneration can only happen once. Alternative systems able to transport greater volumes of healing agent include embedding the healing agent in hollow fibres^13,14^, or biomimetic development based on micro-vascular networks^15,16^ enabling its long-range transport. The goal for self-healing systems is to work towards true biomimetic healing by incorporating a circulatory system that continuously transports the necessary healing products to the damage site. Current state of the art in ultra-fast X-ray tomographic microscopy would enable to watch and follow the healing process in 3D in such systems, ultimately for its optimization, with increased time resolution. For example, time resolved studies collecting more than 200 tomography scans per second (over a period of one minute) can be performed with voxel sizes of ~5 µm^17^. X-ray projection data for a single tomography scan can now be routinely acquired in less than 10 s and with voxel sizes of ~1 µm using synchrotron X-ray sources (e.g.^9^).

Consequently, understanding the cracking process and optimizing the microcapsule density is critical to the self-healing process. Here we have seen that microcapsules within approximately 400 µm either side of the crack plane are ruptured and contribute to the healing process. The observation of small regions of unfilled crack local to some of the capsules could thus be related to the crack face separation and the exhaustion of solvent due to progressive crack opening. In our case we have shown that release of solvent to repair the crack can continue to take place as the crack grows and progressively widens.

## Methods

### Materials

The self-healing epoxy in this study comprises a microencapsulated reactive epoxy resin and solvent that swells the epoxy matrix and enables the resin to react with residual amines in the matrix^6,18^. Urea-formaldehyde microcapsules containing EPON 862 (diglycidyl ether of bisphenol-F) resin dissolved in ethyl phenylacetate (EPA) solvent were manufactured using the emulsion *in situ* polymerization microencapsulation method^3,19^. The stirring rate applied to the reaction mixture, in this case approximately 450 rpm, determined the microcapsule size, while microcapsules were isolated by vacuum filtration. The average microcapsule diameter was 155 µm (standard deviation: 61 µm), determined subsequently from the segmented CT data. Samples for testing were prepared using EPON 828 epoxy resin cured with 12 parts DETA (diethylenetriamine) curing agent per 100 parts of resin, with a prescribed concentration of the microcapsules and the catalyst mixed into the resin. The epoxy mixture was degassed and cured for 24 h at room temperature followed by 24 h at 30 °C. A complete description of the fabrication process for the self-healing material is described elsewhere^3–5^.

### Mechanical testing

**Figure 5:**
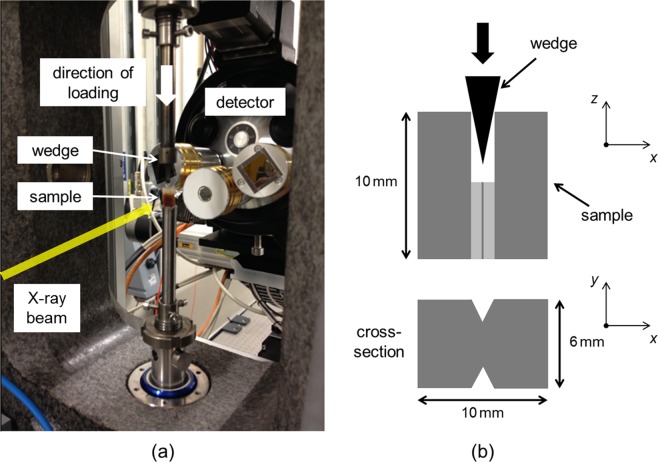
Experimental setup for *in situ* X-ray tomographic microscopy. (**a**) A photograph showing the sample within the mechanical testing rig on the end-station of the I13 imaging beamline, Diamond Light Source. The frame of the rig remains stationary while the sample and loading wedge rotate together as the X-ray projections are collected. (**b**) Schematic showing geometry and form of the sample.

The experimental setup is shown in Fig. 5. In order to observe and follow capsule activation during crack growth a mechanical testing rig (P2R^20^) was used *in situ*, as shown in the experimental setup in Fig. 5a. Small samples for measuring *in situ* crack growth were prepared with geometry 10 × 6 × 10 mm^3^ (*l* × *w* × *h*) as illustrated in Fig. 5b. Side grooves ensured crack growth along the centerline of the sample. A razor blade was used to make a small cut in the epoxy matrix to give a small sharp pre-crack at the notch tip, such that the crack was grown in a controlled manner through the controlled displacement of a wedge into the notch. A CT scan was acquired prior to loading (see Methods: X-ray microtomography). The wedge, fixed to the upper platen of the rig, was lowered slowly into the notch in the sample, itself sitting on the lower platen, with a displacement rate of 10 µm/min and in small steps of 10 µm displacement. Due to the desire to capture time lapse CT measurements during crack growth, this speed was found to be slow enough to control growth of the crack in small steps. Once the wedge was in contact with the sides of the notch, observed by monitoring a small force reading of ~2 N, the wedge was lowered continuously whilst monitoring both the force and a real-time X-ray radiography of the sample. Used together these tools provided the means to follow crack initiation and growth, the real-time radiography proving particularly useful in monitoring the progress of the crack through the movement of fluid from capsules activated along the crack path. Once a crack had initiated, corresponding to a force of ~20 N, an X-ray CT scan was recorded (step 1). Upon further crack growth another CT scan was acquired (step 2). After the second crack growth step the crack had grown to cover almost the full vertical extent of the field-of-view of the imaging setup. The wedge was kept in position during CT scanning for the crack growth steps. The time taken for the test was approximately 2 hours; 30 min for each of the three CT scans (initial state and after each of two crack growth steps) and approximately 15 min for growing the crack during each of the crack growth steps themselves.

### X-ray microtomography

X-ray microtomography measurements were carried out at Diamond Light Source on the Diamond-Manchester Imaging Branchline I13-2, using a monochromatic X-ray beam (energy 13 keV and bandwidth of 0.1 eV). This enabled in-line phase contrast imaging with a parallel-beam setup where attenuation and refractive effects are decoupled to image interfaces between materials of similar attenuation^21^. Projection images were collected using a pco.4000 CCD camera (pixel size 11 µm × 11 µm) coupled with a 4 × objective lens. A further 2 × magnification given by the beamline setup (total magnification of 8×) gave an effective pixel size of 1.12 µm (2.24 µm scaled with 2 × binning) and a maximum field of view of 4.5 mm × 3.0 mm. A single scan comprised 1800 projections around a 180° rotation of the sample with an exposure time per projection of 1 s. Reconstruction of the set of normalized projections was performed using a filtered backprojection algorithm^22^ to produce 3D volumetric datasets of X-ray attenuation consisting of a sequence of evenly spaced slices of thickness 1 pixel. For image analysis and interpretation, a single-distance phase backpropagation algorithm^23^ was applied to the projections to reduce interference fringe effects and to enhance contrast between the matrix material and the capsule contents. In order to quantify the filled/healed crack and the filled capsules a 3D median filter, with a 26-voxel neighbourhood, was first applied to the original X-ray tomographic reconstructions to reduce the background noise. The different material components in each tomographic slice were then segmented using greyscale thresholding. Image processing, segmentation and visualization were performed using Avizo software (from ThermoFisher Scientific).

## References

[1] Hager MD, Greil P, Leyens C, van der Zwaag S, Schubert US (2010). Self-healing materials. Adv. Mater..

[2] van der Zwaag S, van Dijk NH, Jonkers HM, Mookhoek SD, Sloof WG (2009). Self-healing behavior in man-made engineering materials: bioinspired but taking into account their intrinsic character. Phil. Trans. R. Soc. A.

[3] White SR (2001). Autonomic healing of polymer composites. Nature.

[4] Brown EN, White SR, Sottos NR (2004). Microcapsule induced toughening in a self-healing polymer composite. J. Mater. Sci..

[5] Brown EN, White SR, Sottos NR (2006). Fatigue crack propagation in microcapsule-toughened epoxy. J. Mater. Sci..

[6] Caruso MM (2007). Solvent-promoted self-healing epoxy materials. Macromolecules.

[7] Maire E, Withers PJ (2014). Quantitative X-ray tomography. Int. Mater. Rev..

[8] Wu SC, Xiao TQ, Withers PJ (2017). The imaging of failure in structural materials by synchrotron radiation X-ray microtomography. Eng. Fract. Mech..

[9] Sloof WG (2016). Repeated crack healing in MAX-phase ceramics revealed by 4D *in situ* synchrotron X-ray tomographic microscopy. Sci. Rep..

[10] Pei R (2017). Crack healing behavior of Cr2AlC MAX phase studied by X-ray tomography. J. Eur. Ceram. Soc..

[11] Mookhoek SD (2010). Applying SEM-based X-ray microtomography to observe self-healing in solvent encapsulated thermoplastic materials. Adv. Eng. Mater..

[12] Rule JD, Sottos NR, White SR (2007). Effect of microcapsule size on the performance of self-healing polymers. Polymer.

[13] Trask RS, Williams GJ, Bond IP (2007). Bioinspired self-healing of advanced composite structures using hollow glass fibres. J. R. Soc. Interface.

[14] McCombe GP, Rouse J, Trask RS, Withers PJ, Bond IP (2012). X-ray damage characterization in self-healing fibre reinforced polymers. Composites Part A.

[15] Toohey KS, Sottos NR, Lewis JA, Moore JS, White SR (2007). Self-healing materials with microvascular networks. Nat. Mater..

[16] Hansen CJ (2009). Self-healing materials with interpenetrating microvascular networks. Adv. Mater..

[17] Garcia-Moreno F (2019). Using X-ray tomoscopy to explore the dynamics of foaming metal. Nat. Commun..

[18] Caruso MM, Blaiszik BJ, White SR, Sottos NR, Moore JS (2008). Full recovery of fracture toughness using a nontoxic solvent-based self-healing system. Adv. Funct. Mater..

[19] Brown EN, Kessler MR, Sottos NR, White SR (2003). *In situ* poly(urea-formaldehyde) microencapsulation of dicyclopentadiene. J. Microencapsul..

[20] Puncreobutr C, Lee PD, Hamilton RW, Phillion AB (2012). Quantitative 3D characterization of solidification structure and defect evolution in Al alloys. JOM.

[21] Cloetens P (1997). Observation of microstructure and damage in materials by phase sensitive radiography and tomography. J. Appl. Phys..

[22] Titarenko V, Titarenko S, Withers PJ, De Carlo F, Xiao X (2010). Improved tomographic reconstructions using adaptive time-dependent intensity normalization. J. Synchrotron Radiat..

[23] Paganin D, Mayo SC, Gureyev TE, Miller PR, Wilkins SW (2002). Simultaneous phase and amplitude extraction from a single defocused image of a homogeneous object. J. Microsc..

